# Concordance of Blood-Based and Normal Tissue-Based Dihydropyrimidine Dehydrogenase (DPYD) Genotyping

**DOI:** 10.1093/oncolo/oyac057

**Published:** 2022-03-24

**Authors:** Cristina Morelli, Vincenzo Formica, Elena Doldo, Silvia Riondino, Michela Rofei, Lorena Vergilii, Giampiero Palmieri, Hendrik-Tobias Arkenau, Mario Roselli, Augusto Orlandi

**Affiliations:** 1 Medical Oncology Unit, Department of Systems Medicine, Tor Vergata University Hospital, Rome, Italy; 2 Anatomic Pathology, Department of Biomedicine and Prevention, Tor Vergata University Hospital, Rome, Italy; 3 Sarah Cannon Research Institute, Cancer Institute, University College London, London, UK

## Abstract

In response to the recently published article by Sharma et al, this letter to the editor presents data about the concordance of blood and normal tissue-based evaluation of *DPYD* genotyping that suggests that pharmacogenetic screening could be contextual to tumor molecular profiling.

We read with great interest the paper by Sharma et al.^1^ reporting data on the risk of treatment-related death in patients with pathogenic DPYD polymorphisms treated with fluoropyrimidine-based chemotherapy.

Although fluorouracil-induced grade 5 adverse events are rare (approximately 1%),^2^ Sharma et al demonstrated that patients who are homozygous for 1 of the 4 main DPYD-gene alterations (c.1129-5923C>G [HapB3], c.1679T>G [*13], c.1905 + 1G>A [*2A], and c.2846A>T) had a 25.6 times increased risk of death when compared with the homozygous wild-type population.^1^

Since 2018, international guidelines recommend DPYD screening before fluorouracil-treatment start, with reduction of the dosage in case of impaired drug catabolism.^3^ Genotyping the main 4 polymorphisms on germline DNA isolated from peripheral blood cells as well as phenotyping enzyme activity by measurement of uracil blood levels are both European Medicines Agency and Food and Drug Administration approved methods.^3-5^ However, some national recommendations, such as those from the Italian Association of Medical Oncology and the Society of Pharmacology (AIOM-SIF), indicate that only the use of blood-based germline testing should be taken into account.^6^ The widespread application of testing remains an issue and, where the testing is available, an additional blood draw for genotyping is needed.

Tissue DNA analysis is recommended for many tumors before treatment start, and in some cases, such as for the evaluation of the microsatellite instability (MSI) status in colorectal cancer, DNA from both the tumor and normal tissue is extracted to increase the reliability of the test.^7^ On this basis, we investigated the possibility of DPYD genotyping on normal tissue DNA extracted from biopsy or surgical material already available for the assessment of MSI status, thus avoiding an additional blood draw and overcoming logistical challenges.

We tested 5 DPYD gene polymorphisms (IVS14 + 1G>A, c.1905 + 1G>A; c.1679T>G, p I560S; c.2846A>T, p. D949V; c.1129-5923C>G, IVS10C>G; c.2194G>A, V732I) both in blood- and normal tissue-derived DNA from consecutive patients candidate to fluorouracil-based treatment and calculated the blood/tissue concordance rate for each polymorphic locus. Standard Realtime Polymerase Chain Reaction (PCR) (Easy PGX ready DPYD—Diatech Pharmacogenetics) was used for the DPYD genotyping. Two dedicated pathologists declared the adequacy of normal tissue availability before testing. Nine colorectal, 2 esophago-gastric junction, and 1 patients with pancreatic cancer were included.

Heterozygosis of rs1801160 polymorphism (c.2194G>A, V732I) was found in 4 out of 12 patients. Evaluation of heterozygous cases on paired blood and tissue samples showed a 100% overall concordance (Pearson’s coefficient = 1) (Table 1). Concordance between blood and tissue-based results was also confirmed for the remaining 8 wild-type homozygous patients. The 5-fluorouracil administered dose was reduced by 15% in patients who were heterozygous. No grade >1 fluoropyrimidine-related adverse events were reported in the entire cohort.

**Table 1.** Results of consecutive pharmacogenetic DPYD evaluations in patients with 12 GI cancer.

Patient number	Primary tumor	Type of tissue specimen (biopsy or surgical material)	DPYD tissue result	DPYD blood result	MSI status (MSS vs MSIH)
1	Colon	Surgical material	omo wt	omo wt	MSS
2	Colon	Surgical material	G2194A hetero g/a (c.2194 G>A; V7321I)	G2194A hetero g/a (c.2194 G>A; V7321I)	MSS
3	Colon	Biopsy	omo wt	omo wt	MSS
4	Colon	Surgical material	omo wt	omo wt	MSS
5	EGJ	Biopsy	omo wt	omo wt	MSS
6	Colon	Surgical material	G2194A hetero g/a (c.2194 G>A; V7321I)	G2194A hetero g/a (c.2194 G>A; V7321I)	MSS
7	Colon	Surgical material	G2194A hetero g/a (c.2194 G>A; V7321I)	G2194A hetero g/a (c.2194 G>A; V7321I)	MSS
8	Colon	Surgical material	G2194A hetero g/a (c.2194 G>A; V7321I)	G2194A hetero g/a (c.2194 G>A; V7321I)	MSS
9	Pancreas	Biopsy	omo wt	omo wt	MSS
10	Colon	Biopsy	omo wt	omo wt	MSS
11	Colon	Surgical material	omo wt	omo wt	MSS
12	EGJ	Biopsy	G2194A hetero g/a (c.2194 G>A; V7321I)	G2194A hetero g/a (c.2194 G>A; V7321I)	MSS

Abbreviations: M, male; F, female; EGJ, esophagogastric junction; GI, gastrointestinal; wt, wild type; hetero, heterozygote; MSS, microsatellite stable;  MSI-H, high microsatellite instability.

In our series the concordant results between blood and tissue samples in DPYD assessment support the use of tissue testing. We propose for patients where adequate normal tissue DNA is available to concomitantly profile this with tumor DNA, thus avoiding an additional blood draw, reducing waiting times and limiting unnecessary logistical challenges.
